# Long-term effects of PM_2·5_ on neurological disorders in the American Medicare population: a longitudinal cohort study

**DOI:** 10.1016/S2542-5196(20)30227-8

**Published:** 2020-10-19

**Authors:** Liuhua Shi, Xiao Wu, Mahdieh Danesh Yazdi, Danielle Braun, Yara Abu Awad, Yaguang Wei, Pengfei Liu, Qian Di, Yun Wang, Joel Schwartz, Francesca Dominici, Marianthi-Anna Kioumourtzoglou, Antonella Zanobetti

**Affiliations:** Department of Environmental Health (L Shi ScD, M Danesh Yazdi PhD, Y Wei MS, Prof J Schwartz PhD, A Zanobetti PhD) and Department of Biostatistics (X Wu MS, D Braun PhD, Y Wang PhD, Prof F Dominici PhD), Harvard T H Chan School of Public Health, Boston, MA, USA; Gangarosa Department of Environmental Health, Rollins School of Public Health, Emory University, Atlanta, GA, USA (L Shi); Department of Data Sciences, Dana-Farber Cancer Institute, Boston, MA, USA (D Braun); Department of Psychology, Concordia University, Montreal, QC, Canada (Y Abu Awad ScD); School of Earth and Atmospheric Sciences, Georgia Institute of Technology, Atlanta, GA, USA (P Liu PhD); Vanke School of Public Health, Tsinghua University, Beijing, China (Q Di ScD); and Department of Environmental Health Sciences, Mailman School of Public Health, Columbia University, New York, NY, USA (M-A Kioumourtzoglou ScD)

## Abstract

**Background:**

Accumulating evidence links fine particulate matter (PM_2·5_) to premature mortality, cardiovascular disease, and respiratory disease. However, less is known about the influence of PM_2·5_ on neurological disorders. We aimed to investigate the effect of long-term PM_2·5_ exposure on development of Parkinson’s disease or Alzheimer’s disease and related dementias.

**Methods:**

We did a longitudinal cohort study in which we constructed a population-based nationwide open cohort including all fee-for-service Medicare beneficiaries (aged ≥65 years) in the contiguous United States (2000–16) with no exclusions. We assigned PM_2·5_ postal code (ie, ZIP code) concentrations based on mean annual predictions from a high-resolution model. To accommodate our very large dataset, we applied Cox-equivalent Poisson models with parallel computing to estimate hazard ratios (HRs) for first hospital admission for Parkinson’s disease or Alzheimer’s disease and related dementias, adjusting for potential confounders in the health models.

**Findings:**

Between Jan 1, 2000, and Dec 31, 2016, of 63 038 019 individuals who were aged 65 years or older during the study period, we identified 1·0 million cases of Parkinson’s disease and 3·4 million cases of Alzheimer’s disease and related dementias based on primary and secondary diagnosis billing codes. For each 5 μg/m^3^ increase in annual PM_2·5_ concentrations, the HR was 1·13 (95% CI 1·12–1·14) for first hospital admission for Parkinson’s disease and 1·13 (1·12–1·14) for first hospital admission for Alzheimer’s disease and related dementias. For both outcomes, there was strong evidence of linearity at PM_2·5_ concentrations less than 16 μg/m^3^ (95th percentile of the PM_2·5_ distribution), followed by a plateaued association with increasingly larger confidence bands.

**Interpretation:**

We provide evidence that exposure to annual mean PM_2·5_ in the USA is significantly associated with an increased hazard of first hospital admission with Parkinson’s disease and Alzheimer’s disease and related dementias. For the ageing American population, improving air quality to reduce PM_2·5_ concentrations to less than current national standards could yield substantial health benefits by reducing the burden of neurological disorders.

## Introduction

Globally, neurological disorders are the leading group-cause of disability and the second leading group-cause of death, posing an urgent and substantial worldwide public health challenge.^1^ Parkinson’s disease and Alzheimer’s disease and related dementias are the most prevalent neurodegenerative diseases.^2^ Worldwide, an estimated 6 million people have Parkinson’s disease and 44 million people have Alzheimer’s disease and related dementias.^1^ The Global Burden of Diseases, Injuries, and Risk Factors Study 2016 analysis estimated that, since 1990, the prevalence of Parkinson’s disease has increased by 145% and Alzheimer’s disease and related dementias have increased by 117%. The prevalence of these conditions is expected to continue to increase due to lengthening life expectancy.^1^ As no cure exists yet for these conditions, the identification of modifiable risk factors, such as environmental exposures, should be a top research priority.

Concern is mounting that air pollution increases the risk for neurological disorders. Emerging evidence has shown that particulate air pollution is associated with impaired cognitive function,^3,4^ accelerated cognitive decline,^5,6^ Parkinson’s disease, Alzheimer’s disease, and dementia.^7–9^ Research suggests that air pollution might contribute to the potential onset of neurodegeneration via mechanisms such as oxidative stress, systemic inflammation, and neuroinflammation, among others.^10–12^ There is also evidence that air pollution might exacerbate disease progression by accelerating these biological pathways or worsening intermediate processes.^13,14^ Therefore, the first hospital admission with a relevant diagnosis code is occurring sooner than expected. Previous studies that used hospital admission data to assess the effect of air pollution exposure on progression of Parkinson’s disease and Alzheimer’s disease and related dementias included populations residing in the southeastern US region,^7,15^ the Ontario province of Canada,^8^ and well monitored urban areas in the northeastern USA.^9^ To the best of our knowledge, no study to date has been done in the whole US population. Previous studies also focused on older data; as air pollution concentrations have been steadily decreasing in the past few decades in the USA although increases have been seen in some regions, it is essential to establish whether these associations persist even at low concentrations. Hence, evidence remains scarce for the health effects of long-term exposure to low amounts of air pollution across the USA, including locations with sparse or no monitoring.

We aimed to investigate the effect of long-term exposure to fine particulate matter (PM_2·5_) on hospital admissions with a Parkinson’s disease or an Alzheimer’s disease and related dementias diagnosis code. We leveraged a nationwide comprehensive dataset integrating highly accurate and well validated high-resolution PM_2·5_ prediction models and health data for all fee-for-service Medicare beneficiaries across the contiguous United States (2000–16). To address the computational challenges, we applied a novel computationally scalable re-parameterised Cox-equivalent Poisson model.

## Methods

### Study design and participants

We did a longitudinal cohort study in which we constructed a cohort including all Medicare-fee-for-service beneficiaries who were aged 65 years or older in the USA from Jan 1, 2000, to Dec 31, 2016, using the Medicare part A data. We obtained the Medicare inpatient hospital claims from the Medicare Provider and Analysis Review files, which include one record per hospital admission. People are eligible to enter Medicare after they turn 65 years of age, and for each beneficiary, follow-up started on Jan 1, 2000, or Jan 1 of the year following entry into the cohort, until first admission with diagnosis codes for each outcome separately (ie, Parkinson’s disease or Alzheimer’s disease and related dementias), death, or the end of the study period, whichever came first. We extracted age, sex, race, postal code (ie, ZIP code) of residence, and Medicaid eligibility for each beneficiary in each follow-up year. Medicaid (which is distinct from Medicare) is a joint federal–state insurance programme that provides health and nursing home coverage to Americans of all ages on low incomes or with disabilities. Medicaid eligibility is a proxy for individual-level socio economic status—ie, a Medicare beneficiary eligible for Medicaid is likely to have lower socioeconomic status. This study was done under a protocol approved by the Human Subjects Committee of the Harvard T H Chan School of Public Health. Written informed consent of individuals was not required due to the nature of the study.

### Procedures

We used International Classification of Diseases (ICD) codes to identify Parkinson’s disease (ICD-9: 332; ICD-10: G20, G21·11, G21·19, and G21·8) or Alzheimer’s disease and related dementias (ICD-9: 331·0, 290; ICD-10: G30·9, and F05) admissions as principal or secondary diagnoses during the study period (appendix p 3). We observed some overlap in diagnoses for Alzheimer’s disease and dementia. We found that 298 461 (24·2%) of 1 233 132 Medicare-fee-for-service beneficiaries with a dementia diagnosis also received an Alzheimer’s disease diagnosis, while of 2 490 431 Medicare-fee-for-service beneficiaries with Alzheimer’s disease diagnoses, 298 461 (12·0%) also received a dementia diagnosis. This overlap in diagnoses probably reflects different classifications in different medical centres, but not diagnostic misclassification, as routinely collected health data have been shown to achieve high positive predictive values.^15^ Therefore, following the literature,^8^ we combined Alzheimer’s disease and dementia into one outcome for the main analysis and treated them separately as a sensitivity analysis. Thus, separate analyses were done for the two outcomes: Parkinson’s disease and Alzheimer’s disease and related dementias.

We obtained daily PM_2·5_ predictions at a 1 km^2^ spatial resolution across the contiguous United States from a well validated ensemble model.^16^ The model included more than 100 predictor variables from satellite data, land-use and meteorological variables, and chemical transport model simulations. The model was calibrated with daily PM_2·5_ concentrations measured at 2156 monitors—data obtained from the US Environmental Protection Agency’s Air Quality System database and IMPROVE monitoring network—and had excellent performance (10-fold cross-validated *r*^2^ of 0·86 for PM_2·5_ predictions across the USA, ranging from 0·77 in the mountainous USA to 0·92 in the eastern Midwestern USA). The technical details, including information on the model validation, have been previously published.^16^ Using daily PM_2·5_ predictions at 1 km^2^ grid cells, we calculated the daily mean PM_2·5_ concentration for each postal code, by averaging the predictions at the grid cells whose centroids fell within the boundary of that postal code. Based on these results, we estimated annual postal code means and assigned the postal code-wide annual PM_2·5_ concentration means to Medicare enrollees according to the postal code of residence and calendar year. In the USA, the mean population per postal code is around 7500. Each postal code can cover a small area in cities but can be larger in rural areas. The median land area of a postal code is around 92 km^2^.

### Statistical analysis

We collected neighbourhood-level socioeconomic status variables, available at county level or postal code tabulation areas level, which have both been associated with ambient air pollution and implicated in neurological health.^17,18^ These variables were derived from the 2000–16 Behavioral Risk Factor Surveillance System, the 2000 and 2010 US Census, and the American Community Survey for each year from 2005 to 2016 (appendix pp 3–4). Region was classified as northeast, southeast, midwest, southwest, and west.

Given the very large dataset, we applied a Cox-equivalent re-parameterised Poisson approach for each of the two outcomes, coupled with parallel computing, to address the computational challenges (eg, inadequate memory size and lengthy computational time) faced by the conventional Cox proportional hazards model. Specifically, we proposed and fit a stratified quasi-Poisson model to estimate associations between the rate of first hospital admissions with neurological-related diagnosis codes (Parkinson’s disease or Alzheimer’s disease and related dementias) and time-varying annual mean PM_2·5_ concentrations. The dependent variable was the count of outcome-related hospital admissions in each follow-up year, calendar year, and postal code location within strata specified by individual characteristics, using the corresponding total person-time of Medicare-fee-for-service beneficiaries as the offset. By stratifying on individual characteristics—ie, sex, race, Medicaid eligibility, and age at study entry in 2-year categories—we allowed for flexible strata-specific baseline rates. Mathematically, this stratified Poisson model is equivalent to a time-varying Cox proportional hazard model under an Anderson-Gill representation (appendix pp 4–5).^19^ Importantly, the Cox-equivalent Poisson models also allowed use of parallel computing techniques that are not available for Cox models, further reducing the computation time. To account for within postal code correlated observations across years, we applied an m-out-n bootstrap method using postal code units to calculate statistically robust CIs.^20^

To adjust for potential confounding, we also included neighbourhood-level socioeconomic status factors in our analyses. To account for potential residual confounding by spatial and temporal trends, we included indicator variables for region and calendar years. We also estimated effects at low concentrations of PM_2·5_, by restricting analyses to the subset of the cohort with annual exposures always lower than the current national standards (ie, 12 μg/m^3^) over the study period (low-exposure analysis). Finally, to evaluate any potential deviations from linearity in the concentration–response curves, we included penalised splines for the PM_2·5_ term in the models.

To identify subpopulations who might be particularly susceptible, we assessed potential effect modification by sex (men *vs* women), race (white people *vs* Black people *vs* other [Asian, Hispanic, American Indian or Alaskan Native, and unknown]), age (≥80 years *vs* <80 years), Medicaid eligibility (dual *vs* non-dual eligibility) as a surrogate for individual-level socioeconomic status, and urbanicity (quartiles of population density), by including interaction terms between these potential modifiers and PM_2·5_. Specifically, we calculated the effect of PM_2·5_ in each category of the effect modifier and assessed significance of the interaction term. We chose the age of 80 years as a cutoff to distinguish the young and middle-old from the old-old.^21^

We did a series of sensitivity analyses to assess the robustness of our results to confounding, inclusion of prevalent cases, potential outcome misclassification, and exposure time window (appendix pp 5–8). Given that these neurode generative diseases are age-dependent, as additional sensitivity analysis we also considered stratification by age at entry using 1-year intervals. To remove potentially prevalent cases, we ran additional analyses excluding anyone who had a first admission for these outcomes in their first 2 years of follow-up and repeated our analyses. As information in Medicare is only available after beneficiaries turn 65 years old, it is possible that some study participants had a Parkinson’s disease or Alzheimer’s disease and related dementias hospital admission before enrolling to Medicare. This sensitivity analysis—excluding cases with an admission during their first 2 years of enrolment—increases the probability that we are capturing the first admission with a related code. To evaluate whether the associations we observed can be attributed to a different outcome also linked to air pollution, we excluded the subset of Parkinson’s disease and Alzheimer’s disease and related dementias cases with the most frequent category of primary discharge codes (ie, circulatory system disease [ICD-9: 390–459; ICD-10: I00–I99]) from analyses. The primary discharge code appeared in 392 588 (41·1%) cases of Parkinson’s disease and 1 323 044 (45·3%) cases of Alzheimer’s disease and related dementias. Additionally, we added a sensitivity analysis restricting cases only to those with primary diagnoses codes for Parkinson’s disease or Alzheimer’s disease and related dementias. Finally, we considered an alternative exposure window with 1-year lag period (ie, using the annual mean exposure during the year preceding the outcome). Considering that chemical composition of PM_2·5_ mass (and thus relative toxicity) can vary markedly among different regions in the USA, we also did a subgroup analysis by region.

The computations of the analyses of this study were done on the Research Computing Environment, which is supported by the Institute for Quantitative Social Science at Harvard University. We used R software, version 3.3.2 for all analyses.

### Role of the funding source

The funder of the study had no role in study design, data collection, data analysis, data interpretation, or writing of the report. The corresponding author had full access to all the data in the study and had final responsibility for the decision to submit for publication.

## Results

Data were analysed for Medicare beneficiaries who were 65 years and older between Jan 1, 2000, and Dec 31, 2016. The full cohort included 63 038 019 individuals living in 39 065 postal codes (table 1). The mean age at entry was 69·9 years (SD 7·2). There were 478·3 million person-years of follow-up for Parkinson’s disease and 473·7 million for Alzheimer’s disease and related dementias (table 2). The total number of first admissions was 1·0 million for Parkinson’s disease and 3·4 million for Alzheimer’s disease and related dementias. The median follow-up was 7 years (IQR 8). Of the Parkinson’s disease cases, 77 016 (7·5%) of 1 033 669 had Parkinson’s disease as the primary discharge diagnosis code and, of the Alzheimer’s disease and related dementias cases, 502 565 (14·7%) of 3 425 102 did. For the cases identified with secondary diagnoses of these conditions, we examined the distribution of primary diagnostic codes and found that the primary conditions were predominantly circulatory system diseases (appendix p 8).

The low-exposure cohort subset included 21 928 573 individuals living in 15 775 postal codes, with a mean entry age of 69·8 years (SD 7·1). For Parkinson’s disease, the total person-years of follow-up was 156·3 million and for Alzheimer’s disease and related dementias, it was 155·1 million. The number of first admissions was 0·3 million for Parkinson’s disease and 0·9 million for Alzheimer’s disease and related dementias (table 2).

**Figure 1:**
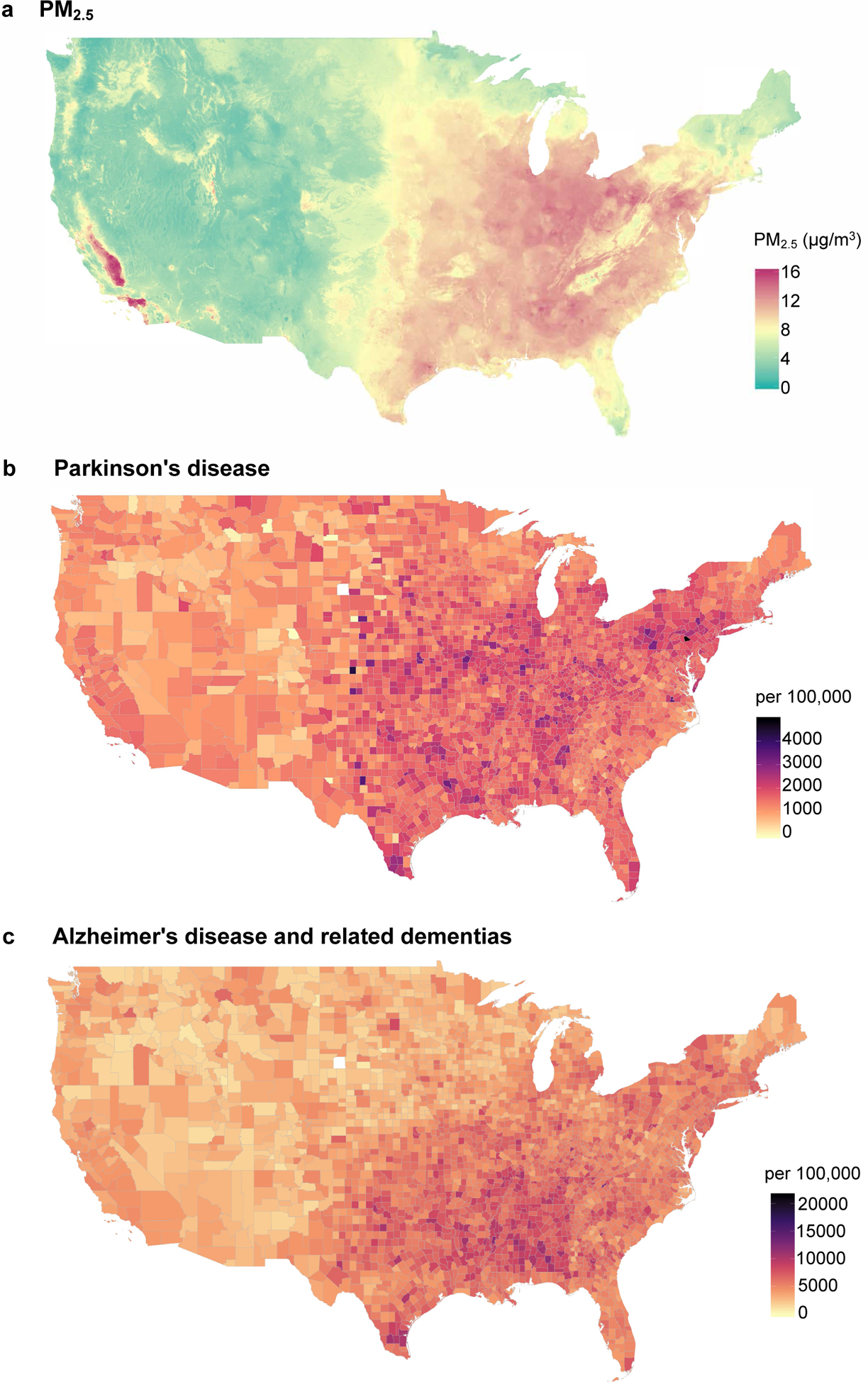
Nationwide concentrations of PM_2·5_, and occurrences of Parkinson’s disease and Alzheimer’s disease and related dementias across the contiguous United States (A) 17-year mean of annual PM_2·5_ concentrations (μg/m^3^). (B) Occurrence of first Parkinson’s disease hospital admissions per 100 000 Medicare beneficiaries. (C) Occurrence of first Alzheimer’s disease and related dementias hospital admissions per 100 000 Medicare beneficiaries (2000–16).

The mean annual PM_2·5_ concentration over the study period was 9·7 μg/m^3^ (SD 3·2, IQR 4·3, 5th to 95th percentile 5·2–15·9; figure 1A). PM_2·5_ concentrations were generally higher in eastern USA than in western USA (except California). Figures 1B and 1C present the occurrence of first hospital admissions with a Parkinson’s disease or an Alzheimer’s disease and related dementias diagnosis code, per 100 000 Medicare beneficiaries across the contiguous United States (2000–16).

Overall, long-term exposure to PM_2·5_ was significantly positively associated with both neurodegenerative outcomes in both the entire cohort and the low-exposure subset. Specifically, in the entire cohort we observed a hazard ratio (HR) of 1·13 (95% CI 1·12–1·14) for Parkinson’s disease admissions and an HR of 1·13 (1·12–1·14) for Alzheimer’s disease and related dementias admissions per 5 μg/m^3^ increase in annual PM_2·5_ concentrations. In the low-exposure subset, we found a slightly elevated association (HR 1·14, 95% CI 1·12–1·16) for Parkinson’s disease and an elevated association (HR 1·18, 1·15–1·21) for Alzheimer’s disease and related dementias admissions per 5 μg/m^3^ PM_2·5_ increase (table 2).

**Figure 2:**
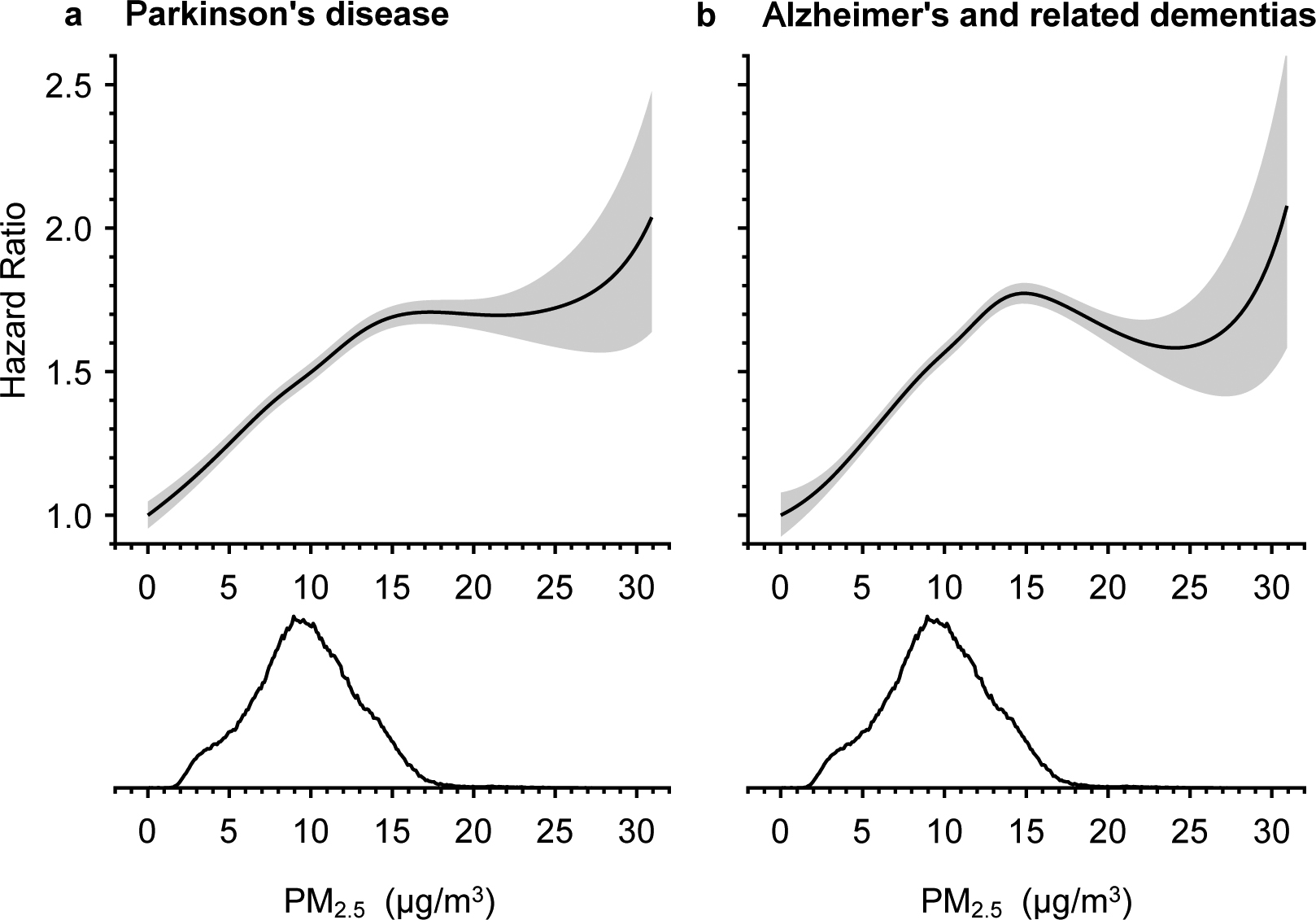
Concentration–response curves of the association between long-term PM_2·5_ exposure and neurological disorders Parkinson’s disease (A) and Alzheimer’s disease and related dementias (B).

Figure 2 shows the concentration–response relationships for Parkinson’s disease and Alzheimer’s disease and related dementias. We observed a strong linear relationship for annual mean PM_2·5_ concentrations less than 16 μg/m^3^, followed by a plateaued association with increasingly larger confidence bands for both outcomes. However, less than 5% of the distribution of the PM_2·5_ concentrations were greater than 16 μg/m^3^.

**Figure 3:**
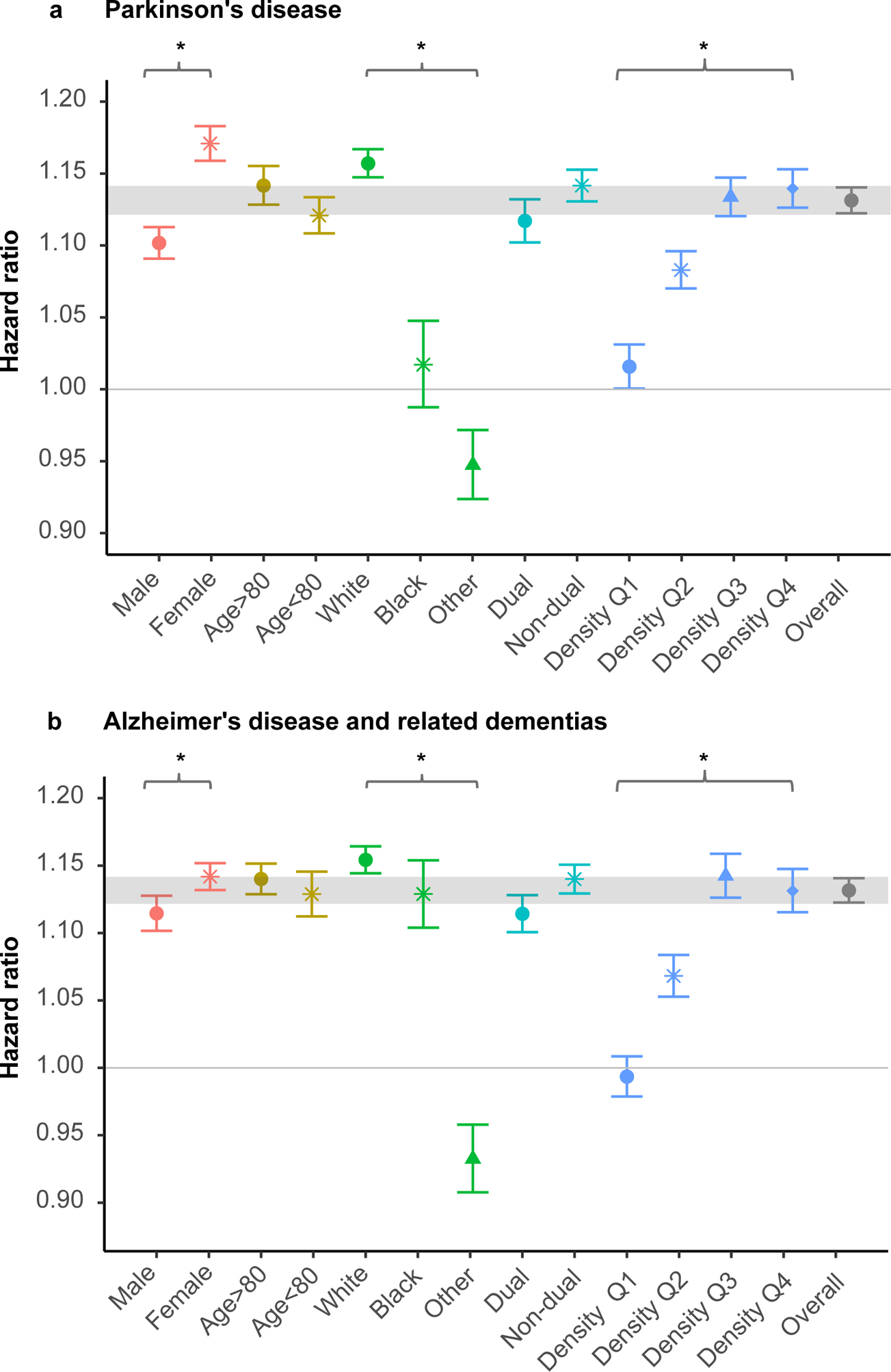
Identification of vulnerable subpopulations Hazard ratios for Parkinson’s disease (A) and Alzheimer’s disease and related dementias (B) associated with a 5 μg/m^3^ increase in PM_2·5_ concentrations by study subgroups. The shading represents the estimated main effects for the overall population. Dual or non-dual refers to eligibility for Medicaid. Density Q1–Q4 denote quartiles of population density—ie, low population density, low to medium population density, medium to high population density, and high population density. Other included Asian, Hispanic, American Indian or Alaskan Native, and unknown race. *Significant modification (at α=0·05 level).

Among the effect modifiers, we found PM_2·5_ effect estimates that were significantly larger in magnitude among individuals in more urban areas versus those in less urban areas (as expressed in quartiles of population density). We also observed higher HRs among those who identified as white than those who identified as Black or Asian, Hispanic, American Indian or Alaskan Native, and unknown, and for women compared with men (figure 3).

For both Parkinson’s disease and Alzheimer’s disease and related dementias, all sensitivity analyses yielded similar results to the main analyses (appendix pp 5–8). When excluding potentially prevalent cases (ie, excluding those who had a first admission in the first 2 years of follow-up), both effect estimates were slightly elevated. The sensitivity analysis in which Alzheimer’s disease and dementia were treated as separate outcomes also yielded significant and positive associations between PM_2·5_ and the two separate outcomes of interest. However, the effect estimates for Alzheimer’s disease (HR 1·17, 95% CI 1·16–1·18) were higher than those for dementia (HR 1·06, 1·05–1·07). Our results were robust to confounding adjustment—ie, the results were almost unchanged when we excluded different sets of covariates in alternative models compared with the main one. Additionally, both exclusion of all cases identified through secondary diagnostic codes and exclusion of those secondary diagnostic cases with circulatory system disease as the primary diagnosis code did not change the main results. Finally, our results were robust to the use of a different exposure window. The 1-year lagged exposure analysis (eg, using annual mean PM_2·5_ in 2005 to link the outcome in 2006) yielded results nearly identical to the findings from our main analysis.

All region-specific results consistently suggested a link between PM_2·5_ and first Parkinson’s disease and Alzheimer’s disease and related dementias hospital admissions, although effect estimates varied by geographical region. In summary, we observed the highest HR for first Parkinson’s disease hospital admission among Medicare enrollees in the northeastern USA and for first Alzheimer’s disease and related dementias hospital admissions in the midwestern USA.

## Discussion

In this large, nationwide prospective cohort of all Medicare-fee-for-service beneficiaries, long-term exposure to PM_2·5_, an indicator for the air pollution mixture at each postal code, was associated with an increased risk of first hospital admission with a Parkinson’s disease or an Alzheimer’s disease and related dementias diagnosis code, even at concentrations less than the current annual national standards (12 μg/m^3^). We also identified women, white people, and more urbanised populations as particularly susceptible subgroups. These findings suggest that improving air quality, with PM_2·5_ concentrations even lower than current national standards, could yield public health benefits.

The shape of the concentration–response relationship between air pollution and neurodegeneration has rarely been assessed in the literature. Only one previous study simply assessed non-linearity using quartiles and found no evidence of deviation.^9^ This result was in agreement with our results, had we used quartiles. Use of splines allowed for a more detailed characterisation of the shape across the PM_2·5_ concentration range. Risk of first hospital admission with a Parkinson’s disease or an Alzheimer’s disease and related dementias diagnosis code, as a proxy for neurodegeneration progression, linearly increased with increasing PM_2·5_ concentrations less than the current annual standards (12 μg/m^3^), suggesting no safe threshold for harmful pollution. Although we detected some deviations from linearity at concentrations greater than 16 μg/m^3^, less than 5% of the observations were higher than that. It is possible that any deviation at such high concentrations could indicate that the flexible penalised splines are sensitive to potential outlying observations with high leverage.

Our findings regarding associations between PM_2·5_ and Alzheimer’s disease and related dementias are consistent with previous research, both in terms of direction and magnitude; of these, one was done in Ontario’s Canadian population,^8^ and the other two were done in regional subpopulations of US Medicare enrollees.^7,9^ Mixed results, including both positive and null findings, however, were reported for the association between PM_2·5_ and Parkinson’s disease in the literature.^9,22,23^ It is worth noting that a comprehensive city-level study in 50 northeastern US cities among Medicare enrollees found higher estimates in magnitude for Parkinson’s disease and Alzheimer’s disease and related dementias than the ones estimated in this study,^9^ which matches our finding of significantly higher PM_2·5_ effects among urban dwellers. Other studies also found similar results in the urban populations they investigated.^7,8^ The observed associations for the other grouping within race are not clear and more work is needed to understand these results. We note, however, that the percentage of the population aged older than 65 years in the USA who are not white or Black is 6·8%.

Both examined diseases have long insidious onsets and the exact timing of disease onset is not known.^24,25^ Furthermore, disease diagnosis probably occurs at a neurologist’s office and not during a hospital admission. Therefore, use of an administrative dataset does not allow investigation of the association between PM_2·5_ and onset of these outcomes. That is, with our analysis we cannot examine true onset incidence or incidence of diagnosis. Our analysis estimates incidence of first hospital admission, which can be interpreted as increased susceptibility to hospital admissions among this patient population and accelerated disease progression. In support of our hypothesis and main findings, the sensitivity analysis excluding people that had a first admission in the first 2 years of the cohort (ie, potentially prevalent cases) resulted in larger in magnitude effect estimates.

In our main analysis, 956 653 (92·5%) of the Parkinson’s disease cases and 2 922 537 (85·3%) of the Alzheimer’s disease and related dementias cases that we identified were based on secondary causes and had a different primary cause of admission. Although this observation was expected, as these outcomes are monitored by neurologists and do not necessarily show up as primary hospital admissions, we were concerned that the observed effect could reflect the signal with the most common primary diagnosis code for these outcomes. Exclusion, however, of Parkinson’s disease and Alzheimer’s disease and related dementias cases with a primary diagnosis for circulatory system disease did not change our results.

Toxicological studies suggest various potential mechanisms via which air pollution might contribute to neurodegenerative progression. Systemic and brain inflammation, for example, enhance the pathogenic alteration of α-synuclein, accelerating the progression of Parkinson’s disease^14^ and Alzheimer’s disease.^13^ Oxidative stress, in addition, is also involved both in initiation and progression, and plays an important role in accelerating Parkinson’s disease progression.^26^ Air pollution might play a key role in neuroinflammation and further exacerbate or initiate dysfunctional protein handling, in the context of amyloid plaques, tau hyperphosphorylation, and neurofibrillary tangles.^27^ Several air pollutants, including PM_2·5_ and ultrafine (<0·1 μm) particulate matter, have been shown to easily cross the blood–brain barrier, providing an important route for air pollutants to interact with the CNS. Indeed, increases in air pollution can elicit increases in the inflammatory response in the prefrontal lobes, with concomitant increases in oxidative damage and amyloid β deposition. The origin of these pathological markers could arise from the direct interaction of air pollutants with microglia in the brain, resulting in a release of pro-inflammatory signals that further facilitate neuronal damage and protein aggregation. Elevation in pro-inflammatory signals can mediate dysfunctional protein handling, in the form of elevations in amyloid β and hyperphosphorylation of tau.^27^ Given the pathogenesis of Alzheimer’s disease and other neurogenerative diseases that are defined by neuroinflammation, oxidative damage, and protein misfolding, exposure to air pollution might serve as an important risk factor in the development and progression of Alzheimer’s disease and Parkinson’s disease pathology and concomitant neurobehavioural deficits. For all neurological outcomes, we observed significantly higher effects of PM_2·5_ among individuals in urban areas versus rural areas. One possible reason might be the abundance of metal-bearing nanoparticles in the urban atmosphere, which have very small diameters and can access the brain directly.^28^ The higher estimates among white people and women could be attributed to a longer life expectancy in these groups—ie, the chance of competing risks among non-white individuals or men is greater, including the probability of death before developing Parkinson’s disease or Alzheimer’s disease and related dementias.^29^

Our study data and methods have several advantages. First, our study population of all Medicare-fee-for-service beneficiaries in the USA gives us ample power to detect effects. This statistical power is particularly useful in environmental studies in which exposures are highly prevalent but effect estimate sizes are often small. Second, our study assessed the whole of the USA, which has greater generalisability than previous smaller cohort studies that were geographically restricted, although our study might not be generalisable to other countries. Furthermore, the aggregation of data into strata of shared individual characteristics not only allowed us to create a more efficient model but also allowed us to analyse a very large dataset with a far smaller computational burden. Given the increase in the use of very large datasets, this novel analytical approach might be useful in other research as well.

Our findings, however, should be interpreted in light of some potential limitations. First, reliance on an administrative cohort did not allow us to examine the relationship between PM_2·5_ and disease onset. Parkinson’s disease and Alzheimer’s disease and related dementias are diseases that do not require hospital admission for diagnosis and treatment; usually, hospital admission occurs at more advanced stages of the disease for treating complications or for adjusting the therapeutic plan. Thus, the hospital admission records cannot represent disease incidence and we probably underestimate the case number when using first hospital admission as a proxy for neurodegeneration. In addition, a positive predictive value of 0·65 for Parkinson’s disease^30^ and about 0·75 for Alzheimer’s disease and related dementias^31^ has been reported when Medicare claims were used, indicating the under-diagnosed nature of neurological conditions using claims records. Furthermore, our results only represent the Medicare-fee-for-service population, which does not include all Medicare beneficiaries. Specifically, earlier in our study period (eg, in 2003), the Medicare-fee-for-service population covered up to 29 230 838 (84·9%) of 34 423 305 Medicare beneficiaries, while in 2016 it was 30 974 063 (65·8%) of 47 099 370 Medicare beneficiaries. It is possible that Medicare-fee-for-service beneficiaries switched to Medicare-HMO (Medicare managed care plan) and back, potentially resulting in some missed cases in our data, as we have no information on Medicare-HMO claims records. Our findings, thus, might not be generalisable to the entire Medicare population. Second, the use of predicted concentrations for exposure assessment might have resulted in some exposure measurement error. However, the prediction model we used is considered to have excellent predictive accuracy,^16^ substantially reducing potential exposure measurement error. In our study, exposure measurement error is likely to be non-differential because the error in the predicted ambient PM_2·5_ concentrations is probably independent of outcome status. Thus, any resulting bias would be towards the null.^32^ Third, we cannot exclude the possibility of potential residual confounding bias. We did, however, adjust all our models for multiple neighbourhood-level socio economic status variables, and thus any potential residual bias is expected to be very small. Individual-level risk factors for neurological disorders, such as smoking, are not available in Medicare. However, we used postal code mean predicted PM_2·5_ to assign exposures, which could only covary with individual-level factors through postal code-level socioeconomic status,^33^ for which we carefully adjusted, thus effectively minimising this potential source of bias. Fourth, our ensemble model predicts total PM_2·5_ mass concentrations, but not all particles have the same toxicity; some studies have shown that traffic-related pollution might be particularly toxic.^34^ Future studies should aim to disentangle specific effects of regional versus local particles.

In conclusion, our study provides strong epidemiological evidence that long-term exposure to air pollution is significantly associated with a higher risk of neurological health deterioration, even at concentrations less than the current national standards. Our findings suggest that policies that result in further reductions in ambient PM_2·5_ concentrations can yield substantial health benefits in the ageing US population, even for those already exposed to low PM_2·5_ concentrations.

## Supplementary Material

Supplemental material

## Figures and Tables

**Table 1:** Cohort characteristics

	Full cohort (n=63 038 019)	Low-exposure cohort (n=21 928 573)
Age at entry, years		
65–74	48 784 857 (77·4%)	17 010 757 (77·6%)
75–84	10 550 039 (16·7%)	3 673 343 (16·8%)
85–94	3 327 268 (5·3%)	1 134 507 (5·2%)
95–104	375 708 (0·6%)	109 934 (0·5%)
105–114	147 (<0·1%)	32 (<0·1%)
Mean (SD)	69·9 (7·2)	69·8 (7·1)
Sex		
Men	28 295 987 (44·9%)	10 084 588 (46·0%)
Women	34 742 032 (55·1%)	11 843 985 (54·0%)
Race		
White	53 229 370 (84·4%)	19 776 603 (90·2%)
Black	5 513 530 (8·7%)	663 313 (3·0%)
Other*	4 295 119 (6·8%)	1 488 657 (6·8%)
Medicaid eligibility		
Eligible	7 853 739 (12·5%)	2 405 354 (11·0%)
Ineligible	55 184 280 (87·5%)	19 523 219 (89·0%)
PM_2·5_ concentration, μg/m^3^	9·7 (3·2)	7·2 (2·3)
Body-mass index, kg/m^2^	27·5 (1·1)	27·3 (1·0)
Ever smoked, %	47·1 (7·7)	48·1 (7·8)
Hispanic, %	9·2 (16·7)	9·2 (16·3)
Black, %	9·1 (17·3)	2·7 (7·5)
Median household income, US$1000	48·0 (21·7)	47·5 (18·9)
Median home value, $1000	159·0 (141·9)	153·9 (131·8)
Below poverty level, %	11·0 (10·9)	9·7 (10·2)
Not graduated from high school, %	28·7 (18·8)	24·2 (17·1)
Owner-occupied housing, %	71·1 (18·8)	75·2 (14·8)
Population density, people per mile^2^	1601·2 (5233·1)	595·1 (1595·8)

Data are n (%) or mean (SD).

* Other included Asian, Hispanic, American Indian or Alaskan Native, and unknown.

**Table 2:** Cause-specific admissions for Parkinson’s disease and Alzheimer’s disease and related dementias, 2000–16

	Parkinson’s disease	Alzheimer’s disease and related dementias
**Main analyses**		
Number of admissions	1 033 669	3 425 102
Total person-years	478 335 593	473 696 618
Median follow-up year	7	7
HR per 5 μg/m^3^ PM_2.5_	1·13 (1·12–1·14)	1·13 (1·12–1·14)
**Low-exposure analyses (<12 μg/m^3^)**		
Number of admissions	301 227	939 035
Total person-years	156 287 478	155 139 930
Median follow-up year	6	6
HR per 5 μg/m^3^ PM_2.5_	1·14 (1·12–1·16)	1·18 (1·15–1·21)

Data are n or HR (95% CI). HR=hazard ratio.

## Research in context

### Evidence before this study

Air pollution is a known risk factor for poorer human health. Concern is mounting that air pollution increases the risk for neurological disorders, the leading group-cause of disability and the second leading group-cause of death according to the Global Burden of Diseases, Injuries, and Risk Factors Study 2016. We searched PubMed and Google Scholar for studies examining associations of air pollution exposure with neurological disorders published from database inception until Aug 25, 2020. We used the keywords: (“PM2.5” OR “fine particulate matter” OR “fine particles” OR “air pollution” OR “air pollutants”) AND (“neurological” OR “neurodegeneration” OR “neurodegenerative” OR “cognitive” OR “Parkinson’s disease” OR “Alzheimer’s disease” OR “dementia”). Although toxicological evidence links long-term PM_2·5_ exposure with adverse effects on the nervous system, the epidemiological evidence remains scarce. Emerging evidence has shown that particulate matter air pollution is associated with impaired cognitive function, accelerated cognitive decline, Parkinson’s disease, Alzheimer’s disease, and dementia. The studies using hospital admission data to look at the effect of particulate matter air pollution on these conditions generally included populations residing in well monitored urban areas, or in a single region of the USA, or in a province of Canada. To date, no study has been done nationwide in the USA. Previous studies also focused on older data; air pollution concentrations in the USA have been steadily decreasing, so it is essential to establish whether these associations persist even at lower concentrations.

### Added value of this study

To our knowledge, this is the first nationwide cohort study of the association between PM_2·5_ exposures and neurodegenerative disease in the USA. Our findings provide strong epidemiological evidence for the association between air pollution and neurological disorders. We showed that long-term PM_2·5_ exposures were significantly associated with an increased risk of first hospital admission with primary or secondary diagnosis codes for Parkinson’s disease and Alzheimer’s disease and related dementias. In addition, we observed that risk of first hospital admission with a diagnosis code for these conditions, as a proxy for neurodegeneration, linearly increased with increasing PM_2·5_ concentrations less than the current national standards (annual mean 12 μg/m^3^), suggesting that no safe threshold exists for health-harming pollution concentrations. One highlight of this paper is that we are leveraging an unparalleled amount of data compared with any previous air pollution study to our knowledge, to provide robust epidemiological evidence with the highest possible scientific rigour. Another key feature is the use of innovative computational approaches to accommodate our very large datasets, which can be applicable to other epidemiological studies that face similar challenges in the era of big data.

### Implications of all the available evidence

Our study adds to the small but emerging evidence base indicating that long-term air pollution exposures are linked to an increased risk of neurological health deterioration, even at PM_2·5_ concentrations less than the current national standards. Our findings suggest that policies that result in further reductions in ambient PM_2·5_ concentrations can yield substantial health benefits in the ageing American population, even for those already exposed to low PM_2·5_ concentrations.
