# Supplementary material for: Long-term effects of PM2·5 on neurological disorders in the American Medicare population: a longitudinal cohort study
Source: Lancet Planet Health. Author manuscript; Available in PMC 2020 Dec 7. (PMC7720425; doi:10.1016/S2542-5196(20)30227-8)
Supplement: Supplemental material [file NIHMS1647889-supplement-Supplemental_material.pdf]

Supplementary Appendix for

Long-term Effects of Fine Particulate Matter on Neurological Disorders in the US Medicare Population: A Nationwide Analysis

Liuhua Shi[1,2]*, Sc.D., Xiao Wu[3]*, M.S., Mahdieh Danesh Yazdi[1], Ph.D., Danielle Braun[3,4], Ph.D., Yara Abu Awad[5], Sc.D., Yaguang Wei[1], M.S., Pengfei Liu[6], Ph.D., Qian Di[7], Sc.D., Yun Wang[3], Ph.D., Prof. Joel Schwartz[1], Ph.D., Prof. Francesca Dominici[3], Ph.D., Marianthi-Anna Kioumourtzoglou[8#], Sc.D., Antonella Zanobetti[1#], Ph.D.

1 ***Department of Environmental Health, Harvard T.H. Chan School of Public Health, Boston, Massachusetts, USA***

2 ***Gangarosa Department of Environmental Health, Rollins School of Public Health, Emory University, Atlanta, Georgia, USA***

3 ***Department of Biostatistics, Harvard T.H. Chan School of Public Health, Boston, Massachusetts, USA***

4 ***Department of Data Sciences, Dana-Farber Cancer Institute, Boston, Massachusetts, USA***

5 ***Department of Psychology, Concordia University, Montreal, Canada***

6 ***School of Earth and Atmospheric Sciences, Georgia Institute of Technology, Atlanta, Georgia, USA***

7 ***Vanke School of Public Health, Tsinghua University, Beijing, China***

8 ***Department of Environmental Health Sciences, Mailman School of Public Health, Columbia University, New York, NY, USA***

**** Drs. Shi and Wu contributed equally to this work***

# Drs. Zanobetti and Kioumourtzoglou are co-senior authors

Corresponding to: Antonella Zanobetti, Department of Environmental Health, Harvard T.H. Chan School of Public Health, Landmark Center-404-M, Boston, MA 02215, USA
Telephone: 617-384-8751 E-mail: azanobet@hsph.harvard.edu

Running title: Effect of Air Pollution on Neurological Disorders

SECTION 1: Details regarding diagnosis of PD, AD, and dementia hospitalization

SECTION 2: Details regarding individual-level and area-level covariates that were adjusted for

SECTION 3: Details regarding the Cox-equivalent approach for massive-scale cohort studies

SECTION 4: Details regarding the sensitivity analyses

SECTION 4.1: Sensitivity analysis regarding exclusion of potentially prevalent cases

SECTION 4.2: Sensitivity analysis regarding confounding adjustment

SECTION 4.3: Sensitivity analysis regarding the categorization of age at study entry

SECTION 4.4: Sensitivity analysis regarding the separate AD and dementia cohorts

SECTION 4.5 Sensitivity analysis regarding the primary diagnostic disease codes

SECTION 4.6 Sensitivity analysis regarding the exposure with 1-year lag period

SECTION 4.7 Subgroup analyses by region

SECTION 5: Details regarding the R codes in this analysis

1. **Details regarding diagnosis of PD, AD, and dementia hospitalization**

We used the International Classification of Diseases (ICD) hospital admission codes to identify persons who were hospitalized with primary or secondary diagnosis billing codes corresponding to Parkinson's disease (PD), Alzheimer's disease (AD) or dementia during 2000-2016. From 2000 up to and including the 3rd quarter of 2015, ICD-9 codes were available, and we used codes beginning with '332·' for PD, codes beginning with '331·0' for AD and codes beginning with '290' for dementia. From the 4th quarter of 2015 onwards, ICD-10 codes were in use therefore we identified codes G20, G21·11, G21·19 and G21·8 for PD, G30·9 for AD and finally, F05 for dementia. To ensure that ICD-10 codes were comparable to the original ICD-9 codes, we compared the difference in incidence proportions from the 3rd to the 4th quarter of 2014 to the 3rd to 4th quarter of 2015. The differences are 0.08% and -0.58% for PD and AD/ADRD (Alzheimer's disease and related dementias), respectively.

2. **Details regarding individual-level and area-level covariates that were adjusted for**

We adjusted for 15 covariates in the main analyses, including four individual-level covariates, eight ZIP code-level variables, two county-level variables, and dummy variables indicating geographical regions. Of the 39,065 ZIP codes where the full cohort resides, 4,551 ZIP codes had missing information on the 15 covariates we considered, which resulted in an exclusion of 2·1% of the Medicare FFS enrollees from our analyses. Details with respect to each of these variables are described below.

Individual-level variables: We acquired four individual-level variables from the Medicare denominator files, such as a 2-year category of age at entry (65 to 66, 67 to 68, 69 to 70, 71 to 72, etc), race (White, Black, or other/unknown), sex (male or female), and an indicator variable for Medicaid eligibility, a surrogate for individual-level socioeconomic status (SES).

ZIP code-level variables: Eight variables available at ZIP Code Tabulation Areas (ZCTA) level were derived from the 2000 U.S. Census, the 2010 U.S. Census, and the American Community Survey (ACS) from 2005 to 2016. If variables were missing for a year, we linearly interpolated or extrapolated their values using available data. The ZCTA-level variables included median home value, median household income, population density, percent Hispanic, percent Black, percent of the population with less than a high school degree, percent below the poverty level, and percent of owner-occupied housing units. These ZCTA-level data were then matched to ZIP codes.

County-level variables: Two county-level variables, average body mass index (BMI) and percent of tht population that were ever smokers, were acquired from the Behavioral Risk Factor Surveillance System (BRFSS) for the period of 2000-2016. These county-level variables were matched to ZIP codes if the ZIP code centroids fell within the county boundary.

Regional dummy variable: We used five geographical regions in the US: Northeast, Southeast, Midwest, West, and Southwest (Figure S1).

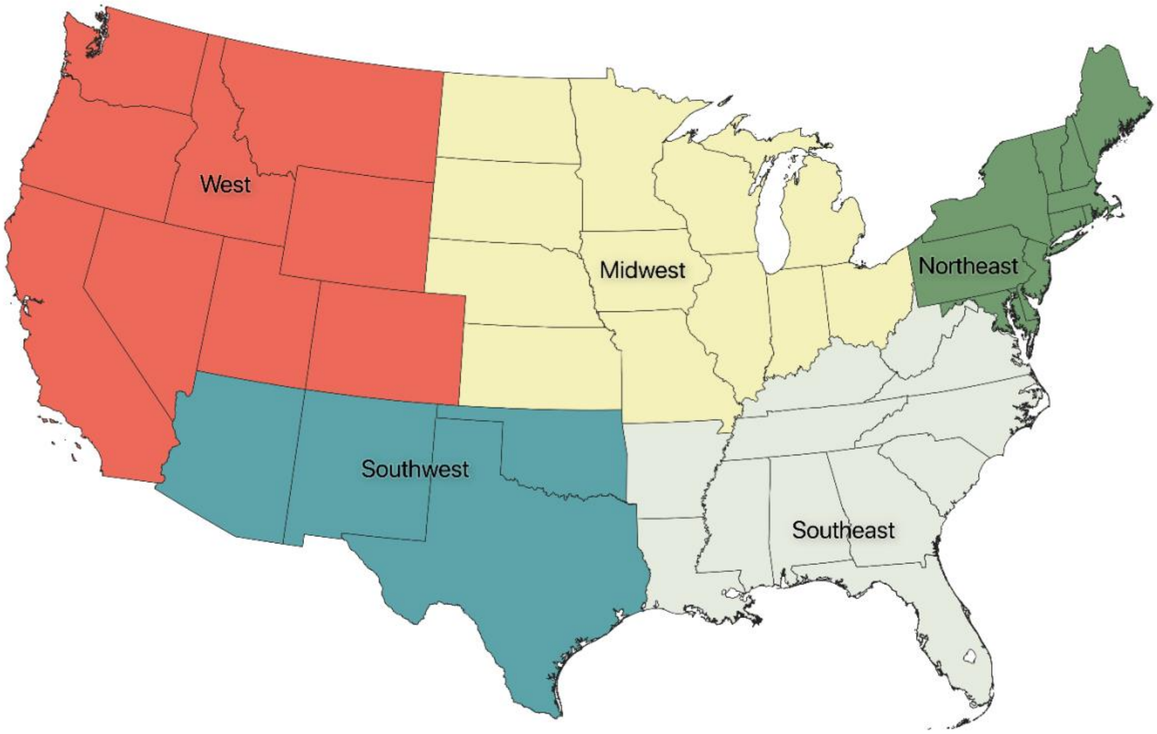

Figure S1. Regional dummy variable

3. Details regarding the Cox-equivalent approach for massive-scale cohort studies

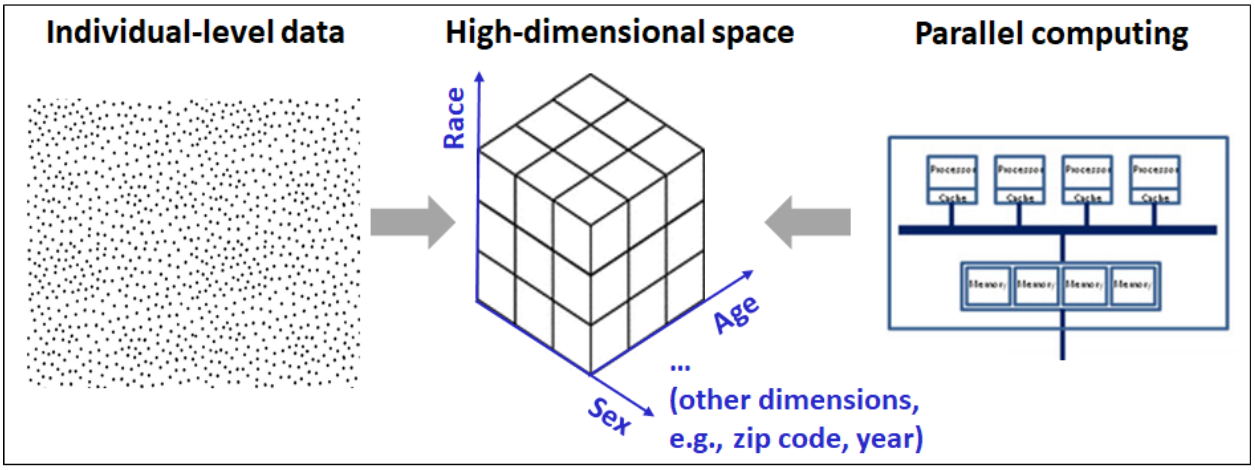

Figure S2. Schematic diagram of the Cox-equivalent approach for massive-scale cohort studies

As illustrated in Figure S2, the key of our approach was to collapse the individual-level records to a high-dimensional space of features, while keeping the integrity of stratum units for analysis (Figure S2). People with the same sex, race, Medicaid eligibility, and 2-year categories of age at study entry, were aggregated and treated as one single grid cell in this high-dimensional space, because they belonged to the same stratum and as such were treated as interchangeable in the analysis. Using this method, we reduced the data size to 13% of the original data size. In addition, the Cox-equivalent Poisson models can utilize parallel computing techniques that are not available for traditional Cox proportional hazard models, further reducing the computational time. Therefore, our approach has the great advantage of overcoming common computational issues (e.g., inadequate memory size and lengthy computational time) and

improving the efficiency of the models when dealing with big data. We used the bam function in the mgcv package version 1·8-29 in R to implement this approach using 24 computing cores in parallel computing. All analyses of this study were conducted on the Research Computing Environment, which is supported by the Institute for Quantitative Social Science in the Faculty of Arts and Sciences at Harvard University.

Below we show the mathematical details on the equivalence between the Cox proportional hazards model and the stratified Poisson model. We consider the following stratified Cox proportional hazards model with Anderson-Gill formulation to examine the long-term effects of $PM_{2.5}$ on the neurological endpoints:

$$h^{c,z}(a,t) = h_0^c(a)exp(\beta_1 W_{z,t} + \beta_2 C_{z,t}) \quad (1)$$

Where $h^{c,z}(a,t)$ denotes the hazard of neurological hospitalization at follow-up year a, calendar year t, and ZIP code z for individual-characteristic strata c (i.e., age group, sex, race, Medicaid eligibility), and $h_0^c(a)$is a strata-specific baseline hazard function. $W_{z,t}$ denotes the annual average $PM_{2.5}$ concentration in ZIP code z in calendar year t. $C_{z,t}$ denotes the ZIP code time-varying covariates in calendar year t and ZIP code z, to adjust for potential confounding.

Model (1) can be written as

$$\frac{E(Y_{a,t}^{c,z})}{T_{a,t}^{c,z}} = h_0^c(a)exp(\beta_1 W_{z,t} + \beta_2 C_{z,t}) \quad (2)$$

Where $E(Y_{a,t}^{c,z})$ denotes the expected number of events at follow-up year a, calendar year t, and ZIP code z for each individual-characteristic stratum c, and $T_{a,t}^{c,z}$ is the corresponding total person-time in that stratum.

Taking the log of both sides, model (2) can be written as

$$log(E(Y_{a,t}^{c,z})) = log(T_{a,t}^{c,z}) + log(h_0^c(a)) + \beta_1 W_{z,t} + \beta_2 C_{z,t} \quad (3)$$

Model (1) is equivalent to the stratified Poisson model (3). The key difference between the two models is that the log-linear model is fitted on an aggregated dataset, yet the Cox model is fitted on individual-level observations. However, since in model (1), both the exposure $W_{z,t}$ and the potential confounders $C_{z,t}$ are at the ZIP code level, we can still construct the aggregated dataset at strata for each ZIP code z, follow-up year a, and calendar year t without losing the capability to detect individual-level health $PM_{2.5}$ effects, assuming the ZIP code average $PM_{2.5}$ exposure is the individual exposure of interest.

4. Details regarding the sensitivity analyses

We conducted a series of sensitivity analyses to assess the robustness of our results. First, we excluded potentially prevalent cases by removing anyone who had a first admission for the outcomes of interest in their first two years of follow-up and repeated our analyses. Second, we fit models at various levels of confounding adjustment by excluding a different set of covariates each time, to assess the bias potential of omitting individual covariates. Third, given that the diseases are age-dependent (e.g., aging is the single greatest risk factor for AD), we considered age at entry in the Medicare cohort using 1-year intervals instead of 2 years for model stratification. Fourth, we repeated the same analysis using AD and dementia outcome separately and we report the individual HRs for these analyses. Lastly, we also looked at the distribution of primary diagnostic codes among cases with secondary PD diagnosis and AD/ADRD diagnosis; circulatory system diseases (CSD) were by far the highest (392,588 [41·1%] and 1,323,044 [45·3%] for PD and AD/ADRD, respectively). To evaluate whether the observed associations could be attributed to an association between $PM_{2.5}$ and CSD, we excluded secondary diagnostic cases with CSD as the primary diagnostic disease and repeated our analyses.

4.1 Sensitivity analysis regarding exclusion of potentially prevalent cases

Because we have no information on the health status of beneficiaries prior to enrollment (i.e., whether they had ever been hospitalized for PD, or AD/ADRD), we repeated our analyses excluding potentially prevalent cases. Specifically, we removed anyone who had a first admission for PD or AD/ADRD in their first two years in the cohort, and repeated analyses with everyone else in. The results show that both effect estimates were slightly elevated, with respective

hazard ratios (HRs) of 1·16 (95% CI: 1·15, 1·17) and 1·17 (95% CI: 1·16, 1·18) for first PD and AD/ADRD, respectively, per 5 μg/m^3 increase in annual $PM_{2.5}$ concentrations.

4.2 Sensitivity analysis regarding confounding adjustment

We fit several alternative models, each excluding a different set of covariates, and compared the effect estimates to assess the impact of potential residual or unmeasured confounding. Table S1 shows the estimated HRs and 95% CIs per 5 μg/m^3 increase in annual $PM_{2.5}$ concentrations for each alternative model.

Table S1. Estimated HRs at different levels of confounding adjustment

	**PD**	**AD/ADRD**
	**HR (95% CI)**	**HR (95% CI)**
Main analysis	1·13 (1·12, 1·14)	1·13 (1·12, 1·14)
Main analysis excluding sex	1·13 (1·12, 1·14)	1·13 (1·12, 1·14)
Main analysis excluding age	1·13 (1·12, 1·14)	1·12 (1·11, 1·14)
Main analysis excluding race	1·13 (1·12, 1·14)	1·12 (1·11, 1·13)
Main analysis excluding Medicaid eligibility	1·14 (1·13, 1·15)	1·14 (1·13, 1·15)
Main analysis excluding time trends	1·17 (1·16, 1·18)	1·14 (1·13, 1·15)
Main analysis excluding dummy region	1·14 (1·13, 1·15)	1·12 (1·11, 1·13)
Main analysis excluding US Census	1·14 (1·13, 1·15)	1·14 (1·13, 1·15)
Main analysis excluding BRFSS	1·14 (1·13, 1·15)	1.16 (1·15, 1·17)

4.3 Sensitivity analysis regarding the categorization of age at study entry

In the main analysis, we considered 2-year categories of age at study entry. We conducted a sensitivity analysis using 1-year age intervals: 65 to 66, 66 to 67, 67 to 68, …, 94 to 95, and above 95 years. This narrower age interval yielded identical results with 2-year age intervals (results not shown) but substantially increased the model run time.

4.4 Sensitivity analysis regarding the separate AD and dementia cohorts

We also constructed cohorts for AD and dementia separately. There were in total 2·5 million and 1·1 million first admissions for AD and dementia, with 475·8 million and 478·6 million person-years of follow-up, respectively (Table S2). For the low-exposure analyses, the corresponding number of first admissions were 0·7 million and 0·3 million, with 155·7 million and 156·4 million person-years of follow-up, respectively. For each 5 μg/m^3 increase in annual $PM_{2.5}$ concentrations, we observed a HR of 1·17 (95% CI: 1·16, 1·18) for AD admissions, and a HR of 1·06 (95% CI: 1·05, 1·07) for dementia admissions. Effect estimates in the low-exposure analyses were both elevated, with corresponding HRs of 1·19 (95% CI: 1·16, 1·22) and 1·19 (95% CI: 1·15, 1·24), respectively.

Table S2. Sensitivity analysis: hazard ratios for Alzheimer's disease and dementia associated with each 5 μg/m^3 increase in $PM_{2.5}$

	AD	Dementia
**Main analyses**		
Number of admissions	2,490,431	1,233,132
Total person-years	475,820,277	478,636,053
Median follow-up year	7	7
HR per 5 μg/m^3 $PM_{2.5}$	1·17 (1·16, 1·18)	1·06 (1·05, 1·07)
**Low-exposure analyses**		
Number of admissions	671,332	340,717
Total person-years	155,722,376	156,446,182
Median follow-up year	6	6
HR per 5 μg/m^3 $PM_{2.5}$	1·19 (1·16, 1·22)	1·19 (1·15, 1·24)

4.5 Sensitivity analysis regarding the primary diagnostic disease codes

We examined the distribution of primary and secondary diagnoses for first hospitalization. For PD, there were a total of 77,016 (7·5%) primary diagnoses and 956,653 (92·5%) secondary diagnoses, whereas for AD/ADRD, there were a total of 502,565 (14·7%) primary diagnoses and 2,922,537 (85·3%) secondary diagnoses. Firstly, we conducted a sensitivity analysis restricting the first hospitalization cases only to those with primary diagnoses codes. For the primary diagnoses analysis, for each 5 μg/m^3 increase in annual PM2.5 concentrations, we observed HRs of 1·14 (95% CI: 1·12, 1·16) and 1·18 (95% CI: 1·16, 1·20) for first hospitalizations with primary diagnosis codes for PD and AD/ADRD, respectively. We are concerned that restricting only to those cases with primary discharge codes may induce bias, as people with these diseases are not commonly hospitalized for these diseases, and therefore the subset of cases with these diseases as primary discharge codes may not be representative of all cases. Reassuringly, the observed HRs are not very different when analyzing cases with primary/secondary discharge codes combined or analyzing cases with primary codes only.

Given the large amount of secondary diagnoses, we compared the distributions of common primary diagnoses among PD and AD/ADRD cases identified through secondary diagnosis vs. non-case Medicare enrollees. The most common primary diagnoses included circulatory system diseases (CSD), cardiovascular disease (CVD), pneumonia, heart failure (CHF), acute myocardial infarction (AMI), stroke, chronic obstructive pulmonary disease (COPD), diabetes and lung cancer. Although the proportions of the primary diagnoses for the common diseases were slightly lower among PD and AD/ADRD cases identified through secondary diagnoses, the rankings were mostly consistent (Tables S3 and S4).

To evaluate whether the observed associations in the main analysis could be attributed to an association between $PM_{2.5}$ and the most common primary diagnosis coding group for PD and AD/ADRD cases identified through secondary diagnosis, we excluded from analysis those cases with CSD as the primary diagnosis and repeated analyses. For each 5 μg/m^3 increase in annual $PM_{2.5}$ concentrations, we observed HRs of 1·11 (95% CI: 1·10, 1·12) and 1·13 (95% CI: 1·12, 1·14) for first hospitalizations with primary or secondary diagnosis codes for PD and AD/ADRD in this subpopulation, respectively. The effect estimates are similar to the main results, indicating that the observed associations cannot be attributed to a $PM_{2.5}$ – CSD association.

Table S3. Sensitivity analysis: distributions of primary diagnostic disease types among PD cases vs. non-cases.

	PD identified from secondary diagnosis	No PD
**Primary Diagnosis n (%)**		
CSD	392,588 (41·1%)	18,137,171 (49·4%)
CVD	268,576 (28·0%)	13,943,584 (38·0%)
Pneumonia	129,339 (13·5%)	5,168,669 (14·1%)
CHF	68,449 (7·2%)	4,482,036 (12·2%)
AMI	52,710 (5·5%)	3,348,458 (9·1%)
Stroke	48,020 (5·0%)	2,063,404 (5·6%)
COPD	40,448 (4·2%)	2,424,983 (6·6%)
Diabetes	25,599 (2·7%)	1,172,902 (3·2%)
Lung cancer	5,435 (0·6%)	809,809 (2·3%)

Table S4. Sensitivity analysis: distributions of primary diagnostic disease types among AD/ADRD cases vs. non-cases

	AD/ADRD identified from secondary diagnosis	No AD/ADRD
**Primary Diagnosis n (%)**		
CSD	1,323,044 (45·3%)	17,064,104 (49·1%)
CVD	858,968 (29·0%)	13,259,384 (38·1%)
Pneumonia	454,323 (15·6%)	4,657,143 (13·4%)
CHF	248,082 (8·5%)	4,252,024 (12·2%)
AMI	187,655 (6·0%)	3,175,249 (9·1%)
Stroke	213,342 (7·3%)	1,853,834 (5·3%)
COPD	33,424 (1·1%)	2,303,755 (6·6%)
Diabetes	96,991 (3·3%)	1,083,020 (3·1%)
Lung cancer	16,051 (0·6%)	809,809 (2·3%)

4.6 Sensitivity analysis regarding the exposure with 1-year lag period

In the main analysis, we use annual average $PM_{2.5}$ during the same year when the outcome occurred. We conducted a sensitivity analysis using the annual average exposure during the year preceding the outcome (e.g., using annual average $PM_{2.5}$ in 2015 to link the outcome in 2016). Since we only access the $PM_{2.5}$ exposures from 2000 to 2016, we constructed a Medicare cohort from 2001 to 2016, and link it to the $PM_{2.5}$ exposure with 1-year lag period. For the 1-year lagged exposure analysis, for each 5 $\mu g/m^3$ increase in annual $PM_{2.5}$ concentrations, we observed HRs of 1·13 (95% CI: 1·12, 1·14) and 1·13 (95% CI: 1·12, 1·14) for first hospitalizations with primary or secondary diagnosis codes for PD and AD/ADRD in this subpopulation, respectively. The results are nearly identical with the findings from our main analysis.

4.7 Sensitivity analysis regarding the subgroup analyses by region

We used five geographical regions in the US: Northeast, Southeast, Midwest, West, and Southwest (Figure S1). To evaluate the potential heterogeneity of associations by geographical regions, we conducted additional analyses, stratified by region of residence. For each 5 $\mu g/m^3$ increase in annual $PM_{2.5}$ concentrations, we observed HRs of 1·26 (95% CI: 1·23, 1·29), 1·06 (95% CI: 1·04, 1·08), 1·15 (95% CI: 1·13, 1·17), 1·12 (95% CI: 1·10, 1·136), and 1·22 (95% CI: 1·19, 1·25) for first hospitalizations with primary or secondary diagnosis codes for PD in Northeast, Southeast, Midwest, West, and Southwest subpopulations, respectively. Moreover, for each 5 $\mu g/m^3$ increase in annual $PM_{2.5}$ concentrations, we observed HRs of 1·06 (95% CI: 1·03, 1·09), 1·21 (95% CI: 1·19, 1·23), 1·27 (95% CI: 1·25, 1·30), 1·09 (95% CI: 1·06, 1·11), and 1·25 (95% CI: 1·22, 1·28) for first hospitalizations with primary or secondary diagnosis codes for AD/ADRD in Northeast, Southeast, Midwest, West, and Southwest subpopulations, respectively. In summary, we observed the highest HR for first PD hospitalization among Medicare enrollees in the Northeast and for first AD/ADRD hospitalization in the Midwest.

5. Details regarding the R code in this analysis

```
#####################################################################
######## Aggregate individual-level data for log-linear model#########
####################################################################
```

```
PD=readRDS(paste0(dir_out,"PD_0016.rds"))
str(PD)
      PD$time_count<-PD$FollowupYearPlusOne-PD$FollowupYear

# Calculate the total count of PD hospitalizations for strata c at
# follow-up year a, time year t and location ZIP code z
PDcount<-aggregate(c(PD[,8]),
by=list(PD$Sex_gp,PD$race,PD$age_gp,PD$Dual_gp,PD$FollowupYear,PD$zipc,
PD$year), FUN=sum)

# Calculate the total person-time at-risk for strata c at follow-up
# year a, time year t and location ZIP code z
time_count<-aggregate(PD[,11],
by=list(PD$Sex_gp,PD$race,PD$age_gp,PD$Dual_gp,PD$FollowupYear,PD$zipc,
PD$year), FUN=sum)

aggregate_data<-merge(PDcount,time_count,by=c("Group.1", "Group.2",
"Group.3", "Group.4", "Group.5", "Group.6", "Group.7"))
colnames(aggregate_data)[8:9]<-c("PD","time_count")
colnames(aggregate_data)[1:7]<-
c("Sex","Race","Age_gp","Dual","FollowupYear","zipc","year")

# Merge with other covariates
covariates=readRDS(paste0(dir_cov,"covariates0016.rds"))
aggregate_PD<-left_join(aggregate_data,covariates, by=c("zipc","year"))

# Save the final dataset
saveRDS(aggregate_PD,paste0(dir_out,"aggregate_PD_0016_2y.rds"))

#######################################################################
############### Cox-equivalent Poisson approach #######################
######################################################################
library("coxme")
library("survival")
library("mgcv")
library("lme4")

print(detectCores())
cl=makeCluster(10,outfile='')
registerDoParallel(cl)

Sys.time()
bam_PD<-bam(PD~pmmean +as.factor(year)+as.factor(region)
            + mean_bmi + smoke_rate + hispanic + pct_blk +
medhouseholdincome + medianhousevalue + poverty + education +
popdensity + pct_owner_occ
+(as.factor(Sex)+as.factor(Race)+as.factor(Age_gp)+as.factor(Dual)+as.f
actor(FollowupYear))^5
+offset(log(time_count)),data=aggregate_PD,family=poisson(link="log"),c
hunk.size=5000,cluster=cl,control=gam.control(trace=TRUE))
```

```
summary(bam_PD)
Sys.time()

########################################################################
#################### m-out-n bootstrapping ############################
#######################################################################
aggregate_PD_boots.list<-split(aggregate_PD, list(aggregate_PD$zipc))
num_uniq_zip <- length(unique(aggregate_PD$zipc))

PD_coefs_boots<-NULL

Sys.time()
for (boots_id in 1:500){
  set.seed(boots_id)
  zip_sample<-
sample(1:num_uniq_zip,floor(2*sqrt(num_uniq_zip)),replace=T)

  aggregate_PD_boots<-
data.frame(Reduce(rbind,aggregate_PD_boots.list[zip_sample]))

  Sys.time()
bam_PD<-bam(PD~pmmean +as.factor(year)+as.factor(region)
            + mean_bmi + smoke_rate + hispanic + pct_blk +
medhouseholdincome + medianhousevalue + poverty + education +
popdensity + pct_owner_occ
+(as.factor(Sex)+as.factor(Race)+as.factor(Age_gp)+as.factor(Dual)+as.f
actor(FollowupYear))^5
+offset(log(time_count)),data=aggregate_PD,family=poisson(link="log"),c
hunk.size=5000,cluster=cl,control=gam.control(trace=TRUE))

  PD_coefs_boots<-c(PD_coefs_boots,bam_PD$coefficients[2])
  Sys.time()
  gc()
}
saveRDS(PD_coefs_boots,paste0(dir_out,"PD_coefs_boots.rds"))

stopCluster(cl)

#bootstrapping standard error:
sd(PD_coefs_boots)*sqrt(floor(2*sqrt(num_uniq_zip)))/sqrt(num_uniq_zip)
```
